# Concurrent Multidomain Cognitive Assessment and Serum BDNF Level in Aged Dogs: Comparison Between Normal Cognition and Mild Cognitive Impairment

**DOI:** 10.3390/ani16172737

**Published:** 2026-09-02

**Authors:** Kittidaj Tanongpitchayes, Areerat Chuasakhonwilai, Kanawee Warrit, Wasana Chaisri, Ravisa Warin, Tanyong Pipanmekaporn, Witaya Suriyasathaporn, Wanna Suriyasathaporn

**Affiliations:** 1 Faculty of Veterinary Medicine, Chiang Mai University, Chiang Mai 50100, Thailand; kittidaj_tanongpit@cmu.ac.th (K.T.); kanawee.w@cmu.ac.th (K.W.); wasana.ch@cmu.ac.th (W.C.); suriyasathaporn.witaya.y3@f.mail.nagoya-u.ac.jp (W.S.); 2 Office of Research Administration, Chiang Mai University, Chiang Mai 50200, Thailand; areeratc.yok@gmail.com; 3 Research Center of Producing and Development of Products and Innovations for Animal Health and Production, Chiang Mai University, Chiang Mai 50100, Thailand; 4 Akkhraratchakumari Veterinary College, Walailak University, Nakhon Si Thammarat 80160, Thailand; ravisa.wa@wu.ac.th; 5 Department of Anesthesiology, Faculty of Medicine, Chiang Mai University, Chiang Mai 50200, Thailand; tanyong24@gmail.com; 6 Department of Biomedical Informatics and Clinical Epidemiology, Faculty of Medicine, Chiang Mai University, Chiang Mai 50200, Thailand; 7 Oversea Campus, Asian Satellite Campuses Institute, Nagoya University, Nagoya 464-8601, Japan

**Keywords:** canine cognitive dysfunction, CADES, cognitive–behavioral test, short-term memory, novel object recognition, biomarker

## Abstract

Early detection of canine cognitive dysfunction syndrome (CDS) is challenged by the limitations of single diagnostic tools, including owner-reported subjectivity and impractical behavioral testing protocols. This study examined a multidomain strategy integrating questionnaires, behavioral testing, and a circulating biomarker to identify measures associated with mild cognitive impairment in aged dogs. A total of 21 aged dogs were classified as cognitively normal (*n* = 9) or mildly cognitively impaired (*n* = 12) using the Canine Dementia Scale (CADES), and they were further assessed based on the Canine Cognitive Dysfunction Rating Scale (CCDR), a battery of behavioral tests across six cognitive domains, and serum brain-derived neurotrophic factor (BDNF) concentrations. The CADES scores differed significantly between the mildly cognitively impaired and cognitively normal groups (median 12.5 vs. 0, *p* < 0.01), whereas the CCDR scores did not (*p* = 0.114). Among the behavioral measures, the delayed match-to-sample task with a 20-s delay and the recognition index derived from novel object recognition testing were significantly associated with cognitive group classification; the other domains showed no significant associations. Serum BDNF concentrations did not differ between the groups or predict group membership. The results of this exploratory study show that targeted behavioral measures may be more strongly associated with mild cognitive impairment than circulating BDNF, thus supporting their potential utility for early cognitive screening in clinical settings. However, these findings should be interpreted with caution, and their diagnostic accuracy should be confirmed in larger cohorts with further validation.

## Simple Summary

As dogs live longer, age-related cognitive decline is becoming an increasingly common problem, yet early detection remains difficult. Owner-based questionnaires can be biased by subjective judgment, and detailed cognitive tests are often impractical in clinical settings. In this study, we combined questionnaires, simple behavioral tasks, and a blood test to detect early cognitive decline in 21 older dogs that were classified as cognitively normal or mildly cognitively impaired. A short-delay memory task and a curiosity-based object test showed potential to differentiate between the two groups, whereas the blood test did not; however, these preliminary results require validation in larger studies. Our findings suggest that simple, practical behavioral tasks may provide veterinarians with a more accessible method for detecting early cognitive decline in aging dogs, thus facilitating the provision of timely care for dogs and their owners.

## 1. Introduction

The population of aged dogs has been steadily increasing in recent years, mainly due to advances in veterinary medicine and veterinary care and increased awareness of dogs’ well-being among pet owners [1,2,3]. As dogs remain a favored companion animal, their longer lifespans have resulted in an increase in age-related conditions, including canine cognitive dysfunction syndrome (CDS). CDS is a neurodegenerative disease that occurs with increasing age and affects older dogs. It shares a pathophysiology with human Alzheimer’s disease, specifically the extensive accumulation of beta-amyloid plaques [4]. The clinical symptoms of CDS are generally seen in dogs aged 7 years or above, but the age of onset can vary between individuals [5,6]. The clinical markers of CDS include behavioral changes such as disorientation; changes in the sleep/wake cycle; changes in social interactions; inappropriate elimination (house soiling); and deficits in cognitive processes such as perception, awareness, learning, and memory [4]. This syndrome not only decreases the quality of life of aging dogs but also places significant emotional, financial, and long-term veterinary care burdens on their owners [6,7]. Therefore, a better understanding of the clinical behaviors and early detection methods of CDS in aged dogs is of paramount importance for veterinary clinics and research studies.

The most common tools for detecting and determining the severity of CDS in clinical practice are questionnaire-based assessments, i.e., the Canine Cognitive Dysfunction Rating Scale (CCDR) and the Canine Dementia Scale (CADES) [8,9,10]. Although these questionnaires are appreciated for their ease of use, low cost, and practicality, they are inherently susceptible to owner subjectivity, as they are based on the owner’s knowledge, frequency of observation, and pattern of interaction with their dog, which can significantly affect reliability [11]. Objective cognitive–behavioral tests examining multiple cognitive domains, such as exploratory behavior, communication, short-term memory, executive function, attention, and novel object recognition, have been used to evaluate cognitive function in aged dogs [12,13]. Such tests provide standardized, individual, and examiner-assessed information and are less prone to observer bias than owner-reported questionnaires, but their clinical use is hindered by the time and specialized equipment required and the inherent variability of behaviors [14,15]. Considering the complementary advantages and specific limitations of questionnaire-based and behavioral assessments, a multidomain approach combining both tools may provide a more comprehensive and objective evaluation of cognitive function in aged dogs.

Peripheral biomarkers have been a promising strategy in human medicine for the early detection and diagnosis of Alzheimer’s disease. In veterinary studies, several biomarkers such as plasma neurofilament light chain (pNfL) and amyloid beta have been investigated for their ability to detect CDS in dogs (10), but their suitability for clinical use remains uncertain. Brain-derived neurotrophic factor (BDNF), a member of the neurotrophin family, is important for learning and memory, as it influences neuronal plasticity, synaptogenesis, and the regulation of neuronal cell survival [16,17,18,19]. Alterations in BDNF levels have been associated with cognitive changes in humans and experimental animals, including age-related cognitive decline, Alzheimer’s disease, and post-operative cognitive dysfunction [19,20,21,22]. However, limited research has investigated the association between BDNF and CDS in aging dogs or whether BDNF levels are associated with performance on cognitive and behavioral tests in early-stage cognitive decline. The Canine Cognitive Dysfunction Syndrome Working Group identified future needs such as the development of blood biomarkers and cognitive testing batteries for routine clinical use in order to improve diagnostic accuracy and therapeutic monitoring [23]. Hence, the quantification of biomarkers, along with comprehensive cognitive and behavioral testing, may provide useful information regarding the diagnosis and progression of CDS in clinical settings.

Thus, in this study, we examined a multidomain assessment strategy to address the limitations of single-tool approaches by simultaneously using both the CCDR and CADES questionnaires to distinguish dogs with normal cognition from those with cognitive impairment and to measure cognitive performance using a battery of behavioral tests spanning six cognitive domains. To further inform the results of the cognitive evaluation, we also measured serum BDNF concentrations. We hypothesized that aged dogs with cognitive impairment would show measurable deficits in specific cognitive domains and reduced serum BDNF concentrations compared to cognitively normal dogs. We further hypothesized that these objective measures would be significantly correlated with questionnaire-based severity scores, thus serving as reliable predictors of cognitive dysfunction. To achieve this, we evaluated the correlations between the evaluation tools and the predictive capacity of the cognitive test results and serum BDNF levels using binary logistic regression, which has the potential to identify reliable markers and useful tools for the evaluation and diagnosis of CDS. Ultimately, the results of this study can contribute to the development of more objective and comprehensive guidelines for the diagnosis of CDS, thus improving the quality of life of aging dogs.

## 2. Materials and Methods

Senior dogs (>8 years old) that were clinically healthy or had a stable concurrent disease status, regardless of sex or breed, were initially recruited from volunteer pet owners through advertisements at the Small Animal Teaching Hospital, Faculty of Veterinary Medicine, Chiang Mai University (FVM CMU), from April 2024 to December 2024. Information regarding dog breed was obtained from owner records. However, breed identification without DNA confirmation (e.g., via owner report or visual inspection) may be subject to inaccuracies [24,25]. Dogs were excluded if they had clinical neurological disorders, an abnormal mental status, blindness, an inability to walk, aggressive behavior, or uncontrolled systemic diseases; if they currently took medications affecting their mental or neurological status or took antioxidant supplements within one week prior to examination; or if their owners were unable to supervise or appropriately interact with them. The final selection was based on criteria regarding the feasibility of conducting cognitive assessments of the dogs, including a non-aggressive temperament, the absence of degenerative diseases that would interfere with cognitive testing, and owners who could comply with study appointments and protocols. None of the dogs recruited in this study met the exclusion criteria. Informed consent was obtained from all dog owners prior to participation. The study protocol was approved by the Animal Care and Use Committee of the Faculty of Veterinary Medicine, Chiang Mai University, Thailand (FVM-CMU-ICUC Ref. No. S4/2023). Ethical review and approval for human participants were waived under CMUREC SOPs 08/V3.1 (Articles 6.1 and 6.2), as the dog owners served only as informants of their animals’ behavior via the CADES and CCDR questionnaires, with no personally identifiable data retained.

On the testing date, the owners were instructed to withhold the dog’s morning meal without water restrictions to minimize variability in food motivation. In addition, baseline food motivation was confirmed for each dog by offering treat samples prior to the formal behavioral assessments. The cognitive assessment consisted of three components: two cognitive questionnaires completed by the pet owners, six domains of cognitive–behavioral testing, and serum BDNF biomarker analysis. The cognitive questionnaires were completed by the owners on the day of the behavioral examination. The behavioral assessments were conducted at the Neurology and Rehabilitation Clinic of the Small Animal Teaching Hospital, FVM CMU, in a dedicated room separate from the main hospital to minimize distractions. During testing, only the owner, the examining veterinarian, and the dog were present. A standardized 3.0 × 2.0-m non-slip mat was used throughout all sessions, with fixed stationing points established for both the examiner and the subject to minimize procedural bias. Two video cameras were used to record each dog’s behavior from straight and top views. Blood samples were collected after completing all cognitive assessments to minimize stress; details of sample processing are described below. To minimize observer bias, all cognitive assessments of the recruited dogs were evaluated at the end of the study phase.

Two validated cognitive questionnaires were selected for this study: The CCDR [8] and the CADES [9]. Both questionnaires were administered by an experienced veterinary neurologist through structured interviews with the owners on the day of the behavioral examination during the dog’s acclimation period. The CCDR assesses 13 behavioral domains, including orientation, memory, apathy, olfactory impairment, and locomotion, and it classifies dogs into three groups: normal (0–39), at risk of cognitive dysfunction (40–49), and cognitive dysfunction (≥50). The CADES evaluates four cognitive domains, namely, spatial orientation, social interaction, sleep–wake cycles, and house soiling, and it categorizes dogs into four severity groups: normal aging (0–7), mild cognitive impairment (8–23), moderate cognitive impairment (24–44), and severe cognitive impairment (45–95). The dogs were subsequently classified into cognitive status groups based on their CCDR and CADES scores; the group distribution is reported in Section 3.

**Figure 1:**
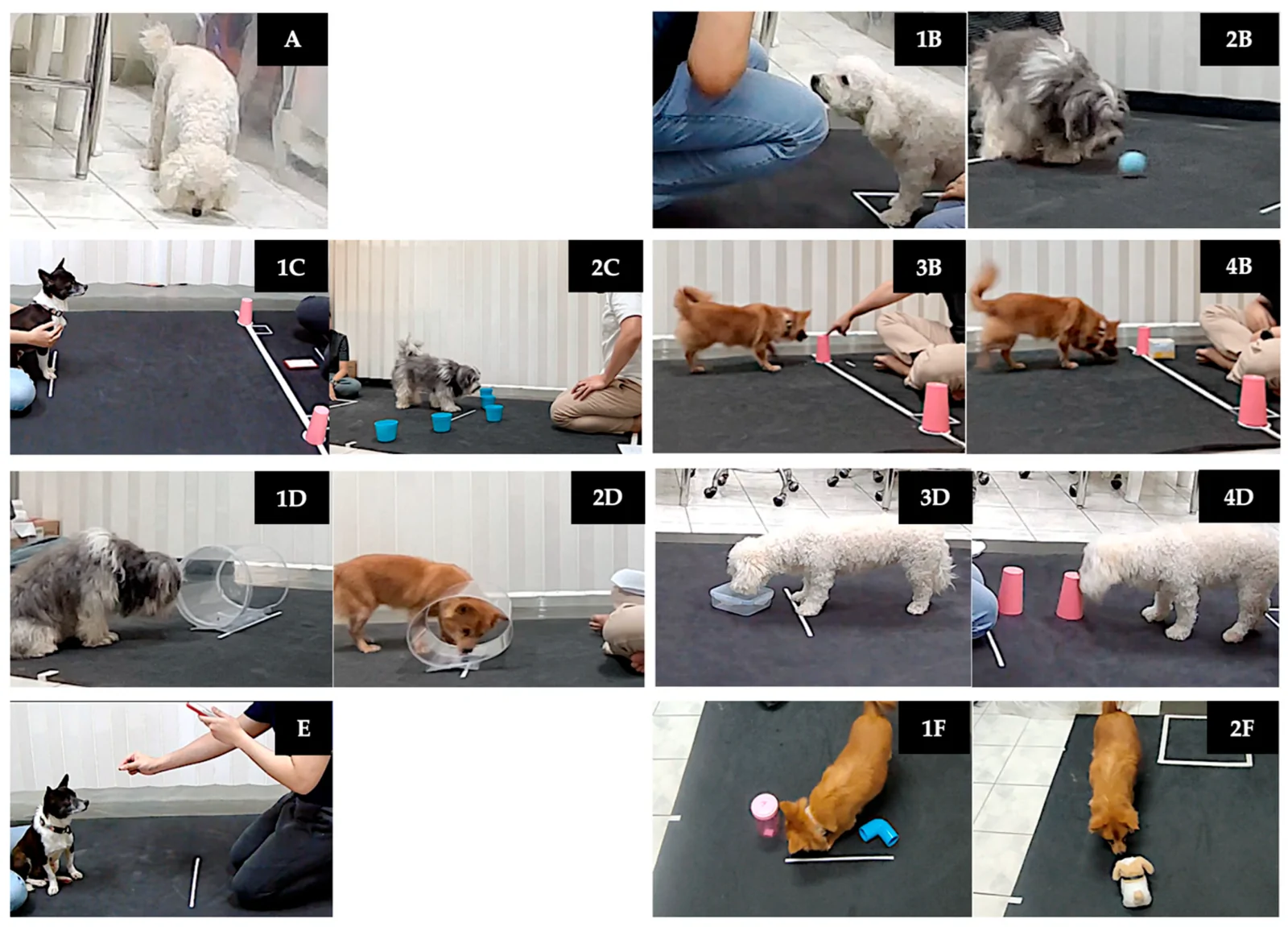
Multidomain cognitive assessment of recruited dogs in a distraction-free clinical environment with a standardized 3.0 × 2.0 m mat. (**A**) Exploratory behavior. Communication, including greeting behavior (**1B**), retrieval task (**2B**), pointing cue (**3B**), and marker cue (**4B**). Short-term memory tasks, including delayed match-to-sample (DMTS) (**1C**) and five pots tasks (**2C**). Executive function assessments, including cylinder task (inhibitory control and detour tasks) (**1D**,**2D**), problem box task (**3D**), and bait task (**4D**). (**E**) Attention. Novel object recognition using neophilia (**1F**) and neophobia paradigms (**2F**).

The cognitive–behavioral test protocol was adapted from previously published studies on CDS [12,13,14,26,27] to assess six cognitive domains of canine cognitive performance: exploratory behavior, communication, short-term memory, executive function, attention, and novel object recognition (Figure 1). An overview of the testing protocols and outcome measures is provided in Table 1, and detailed scoring criteria are presented in Table 2. Step-by-step descriptions of each cognitive–behavioral test are available in Supplementary Material S1. All behavioral assessments were performed by a single experienced evaluator (K.T.). To ensure high intra-rater reliability over time, the evaluator strictly adhered to standardized definitions and objective scoring criteria, which minimized subjective bias throughout the study. Prior to testing, each dog was allowed an acclimation period of at least 30 min in the examination room with its owner and the examiner, without direct interaction, to reduce novelty-induced stress. Subsequently, a vision screening was performed to confirm adequate visual function, as blindness was an exclusion criterion for this study. The cognitive–behavioral tests took approximately 2.5–3 h to complete, depending on the dog’s behavior and compliance.

Serum BDNF levels were quantified using a commercial ELISA kit (Invitrogen^®^ Canine BDNF ELISA Kit, Cat#EC1RB, Invitrogen; Thermo Fisher Scientific, Waltham, MA, USA). To this end, 3 mL blood samples were collected from either the cephalic or saphenous vein using a 3 mL syringe with a 23-gauge needle, following the preparation of the venipuncture site with 70% alcohol. The collected blood was immediately transferred into a plain vacutainer tube for serum separation. During the procedure, the dogs were gently held using standard manual restraint to ensure safety and minimize stress. The blood samples were kept at room temperature for exactly 60 min before serum separation via centrifugation at 1000 rpm for 15 min. The separated sera were stored in Eppendorf tubes at −80 °C until analysis, which was performed within six months of collection. Serum BDNF concentrations were determined according to the manufacturer’s instructions.

### Statistical Analysis

Statistical analyses were performed using IBM SPSS Statistics for Mac (Version 30.0; IBM Corp., Armonk, NY, USA) and RStudio (Version 4.4.1; RStudio, Inc., Boston, MA, USA). Statistical significance was set at *p* < 0.05. Descriptive statistics, such as means and percentages, were used to summarize the signalments and outcomes of the recruited dogs. Data normality for continuous variables was assessed using the Shapiro–Wilk test. As serum BDNF concentrations were not normally distributed, a natural logarithm (log) transformation was applied prior to all parametric statistical analyses involving BDNF. Differences in continuous data (e.g., log-transformed serum BDNF levels and percentages of correct choices in cognitive tests) between dogs with normal cognition and those with cognitive impairment were evaluated using independent *t*-tests or Mann–Whitney U tests, as appropriate. Differences in ordinal data, including the cognitive questionnaire and ordinal cognitive test scores (e.g., 4- or 5-point scales), were explicitly analyzed using the Mann–Whitney U test. Nominal categorical data, such as binary outcomes, were analyzed using Fisher’s exact test. Correlations involving cognitive test outcomes, cognitive questionnaire scores, and log-transformed serum BDNF levels were analyzed using Pearson’s correlation or Spearman’s rank correlation coefficients. To evaluate cognitive test outcomes and serum BDNF levels (log-transformed) as predictors of cognitive impairment, univariable logistic regression was initially conducted. To account for multiple hypothesis testing across the univariable logistic regression comparisons, we applied the Benjamini–Hochberg procedure to control the false discovery rate (FDR), with *q* < 0.05 considered significant. Subsequently, multivariable logistic regression was conducted to adjust for potential confounders using a forward selection method. Following this approach, variables were allowed to freely enter and remain in the final model only if they met the criterion of *p* < 0.05. Data were visualized using GraphPad Prism 9 (GraphPad Software, San Diego, CA, USA).

## 3. Results

### 3.1. Animal Grouping and Cognitive Questionnaire Scores

A total of 21 senior dogs were recruited, comprising several breeds, including Poodles (*n* = 6), mongrels (*n* = 6), Shih Tzus (*n* = 3), and Pomeranians (*n* = 3), along with single representatives of Miniature Schnauzers, Chow Chows, and Chihuahuas. In this study, the CADES classified the recruited dogs into two groups: dogs with normal cognition (*n* = 9) and those with mild cognitive impairment (*n* = 12). However, no subjects reached the threshold for moderate or severe cognitive impairment. In contrast, the CCDR classified all recruited dogs as having normal cognition (*n* = 21). Therefore, for further analysis, the dogs recruited in this study were categorized according to the CADES as cognitively normal dogs (*n* = 9) and mildly cognitively impaired dogs (*n* = 12). The signalments and baseline characteristics of the two groups are summarized in Table 3. Statistical analysis confirmed that there were no significant differences between the groups at baseline (*p* > 0.05). A comparison of the log-transformed serum BDNF levels in picograms per milliliter revealed that they did not differ between the cognitively impaired and cognitively normal dogs (*p =* 0.915).

### 3.2. Comparison of Cognitive Test Outcomes Between Normal Cognition and Cognitive Impairment Groups

A comparison of the cognitive test outcomes of cognitively normal and cognitively impaired dogs is shown in Table 4. Notably, all dogs in both groups successfully completed the vision screening and the problem box task in Trial 1 (100% success rate). The results of the short-term memory and neophilia tests showed significant associations with cognitive status. Regarding the short-term memory test, the percentage of correct choices in the DMTS task with a 20-s delay was significantly lower (*p* = 0.034) in the cognitive impairment group than in the normal cognition group. Cognitively impaired dogs performed worse on the DMTS task with a 20-s delay than cognitively normal dogs. Regarding the neophilia test, the recognition index of the cognitively normal dogs (RI = 0.76) was higher than that of the cognitively impaired dogs (RI = 0.26), and this result was statistically significant (*p* = 0.031).

### 3.3. Correlation Between Cognitive Group, Questionnaire Scores, and Cognitive Test Outcomes

**Figure 2:**
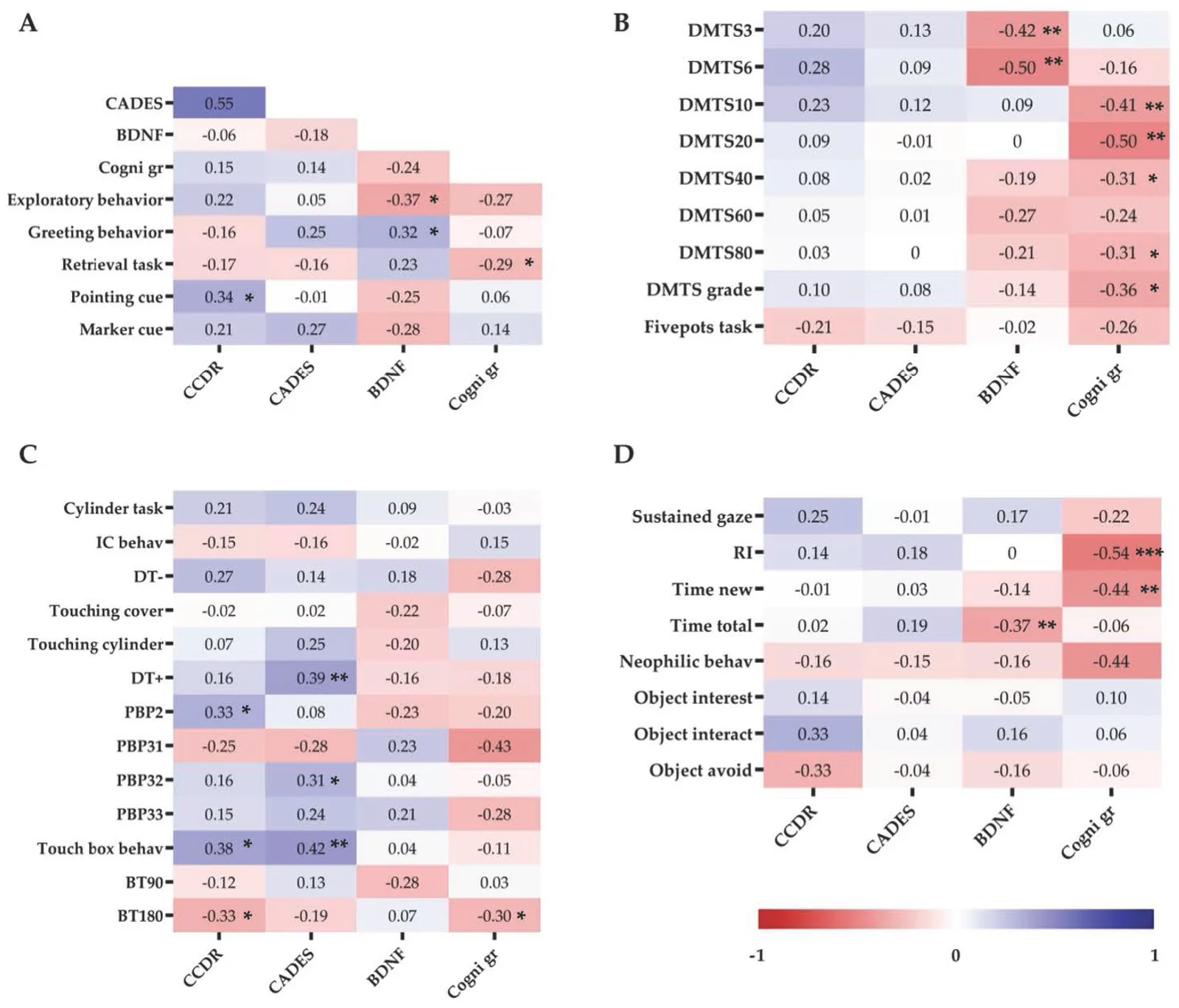
Correlation heatmaps illustrating the relationships between clinical parameters (CCDR score, CADES score, serum BDNF level, and cognitive group) and dogs’ cognitive test outcomes across six domains: (**A**) exploratory behavior and communication; (**B**) short-term memory; (**C**) executive function; and (**D**) attention and novel object recognition. Numbers within the boxes represent correlation coefficients. Asterisks indicate statistical significance: * *p* < 0.1, ** *p* < 0.05, and *** *p* < 0.01. Abbreviations: CCDR, Canine Cognitive Dysfunction Rating; CADES, Canine Dementia Scale; BDNF, brain-derived neurotrophic factor; Cogni gr, cognitive group; behav, behavior; DMTS, delayed match to sample (numbers indicate delay in seconds, e.g., DMTS3 = 3-s delay); IC, inhibitory control; DT, detour trial; DT+, enter from detour side; PBP2, food-obtainable trial; PBP31, block trial (looking at owner); PBP32, block trial (looking at experimenter); PBP33, block trial (orienting toward the box); BT90, bait task (changing 90°); BT180, bait task (changing 180°); RI, recognition index; Time new, time spent with novel object; Time total, total time spent; Prop interact, proportion of interaction; Prop avoid, object avoidance.

Figure 2 presents a correlation heatmap of the CCDR score, CADES score, serum BDNF level, cognitive group, and cognitive test outcomes across six domains for all recruited dogs. The recognition index (r = −0.54) and time spent with a novel object (r = −0.44), derived from the neophilia test (Figure 2D), showed significant negative correlations with the cognitive group. The delayed match-to-sample (DMTS) tasks at 10 s (r = −0.41) and 20 s (r = −0.50) (Figure 2B) also showed significant negative correlations with the cognitive group. Only the CADES scores showed significant correlations with the cognitive test outcomes, whereas the CCDR scores showed no correlations with any outcome. Positive correlations were observed between DT+ in the detour task (r = 0.39) and touching behavior in the problem box task (r = 0.42) with increasing CADES scores (Figure 2C). Serum BDNF levels showed significant negative correlations only with the DMTS task at 3 s (r = −0.42) and 6 s (r = −0.49) (Figure 2B), with no correlations observed for the other cognitive tests.

### 3.4. Univariable Binary Logistic Regression Analysis of Cognitive Group Classification

The results of the univariable binary logistic regression analysis of cognitive group classification (normal cognition or cognitive impairment group) according to cognitive performance outcomes are summarized in Table 5. The analysis showed that dogs with lower percentages of correct choices in the delayed match-to-sample (DMTS) task with a 20-s delay were more likely to have a cognitive impairment (OR = 0.98, 95% CI: 0.95–1.00, *p* = 0.048). Similarly, a lower recognition index on the neophilia test was significantly associated with cognitive impairment (OR = 0.04, 95% CI: 0.002–0.761, *p* = 0.032). No significant associations were found for serum BDNF levels or any other cognitive test variables. To address the potential risk of false-positive findings from multiple comparisons, false discovery rate (FDR)-adjusted *p*-values (q-values) for the univariable binary logistic regression were calculated and are provided in Supplementary Table S1. The results of the multivariable logistic regression analysis showed that the recognition index (RI) was the only independent variable that met the criteria for entering and remaining in the final model (adjusted OR = 0.042, 95% CI: 0.002–0.742, *p* = 0.030), which is consistent with the findings of the univariable analysis.

## 4. Discussion

The use of multidomain assessments is important for improving the objective diagnosis of cognitive decline in aged dogs. In the present study, we investigated which specific cognitive tests and biological markers, when combined with the CADES—a validated, owner-reported screening tool widely adopted in clinical veterinary practice [9]—showed a significant association with classification into the mild cognitive impairment group. Our exploratory findings indicate that specific behavioral tests, including the DMTS task and the novel object recognition test (which provides the recognition index), may be more strongly associated with cognitive impairment than circulating biomarker analysis, as serum BDNF concentrations neither differed between groups nor predicted group membership. Together, these exploratory results highlight specific cognitive tests as promising candidates for future validation in the early detection of canine cognitive decline in clinical practice.

In our cohort, senior dogs with cognitive impairment had a significantly higher CADES score than those with normal cognition, but no significant difference was detected between CCDR scores, despite age matching. Interestingly, all dogs identified as having a mild cognitive impairment by the CADES were below the diagnostic cut-off for cognitive dysfunction syndrome on the CCDR. This difference is directly related to the structural focus of each tool. The CCDR contains 13 behavioral dimensions and is better suited for identifying severe cognitive dysfunction, while the CADES has four specific domains and can determine the severity of cognitive impairment (mild, moderate, or severe) [8,9,10]. Similarly, previous research has shown that the CADES is more sensitive to mild-to-moderate cognitive changes, whereas the CCDR may fail to detect these early-stage cognitive changes [4,9,11]. The observation that cognitively impaired dogs were detected only by the CADES suggests that these dogs were in the early (mild) stages of decline. Combined with the clinical screening conducted to rule out other medical conditions, this provided a reliable basis for our group classification and supports the use of the CADES as a sensitive tool for early screening.

The DMTS task was conducted to evaluate short-term memory, and the results show that a 20-s delay is a critical interval for discriminating between normal cognition and mild cognitive impairment. While performance in shorter intervals did not show significant differences, dogs with mild cognitive impairment selected the correct location significantly less frequently, specifically at the 20-s time point. This finding is consistent with the results of a study performed by Zanghì et al. (2015), where the authors reported a significant difference in the performance of dogs with early cognitive decline in the 20-s delay interval [28]. The particular delay intervals that we chose allowed for a more fine-grained characterization of early memory impairment than prior studies that looked at performance across longer delay intervals [13]. The results of a correlation analysis and binary logistic regression showed that the 20-s delay was sensitive and may be a useful screening threshold in clinical settings. Interestingly, while the 10-s delay was significantly correlated with cognitive status, it did not provide predictive power in the binary logistic regression, suggesting that dogs retain sufficient memory capacity during early-stage decline that allows them to successfully navigate short delays (e.g., 10 s), underscoring the importance of the 20-s interval to reliably detect mild cognitive impairment.

Beyond short-term memory deficits, our assessment of the neophilia task showed a significant decrease in the recognition index, i.e., the proportion of time spent exploring a novel object, in mildly impaired dogs compared to normal aged controls. This negative correlation with cognitive status was further supported by binary logistic regression, highlighting the ability of the recognition index to discriminate dogs with mild cognitive impairment from their cognitively normal counterparts. Our results extend the findings of previous studies [14,29] reporting a reduction in neophilia and recognition index scores in older dogs compared to younger controls, and they show that this deficit is particularly evident in the early stages of cognitive decline in an age-matched cohort. Mechanistically, this reduced exploratory drive can be explained by a decline in recognition memory, a key cognitive domain that naturally drives animals to attend to novel stimuli over familiar ones in order to adapt to new environments [30,31]. Because neophilia reflects a dog’s capacity to interact with its surroundings and acquire new behaviors [32], its decline serves as a distinct behavioral marker of early cognitive aging. Accordingly, the recognition index derived from the neophilia task may serve as a practical behavioral measure to support the early detection of mild cognitive impairment in clinical veterinary settings. Although the DMTS task and the RI were significantly associated with cognitive group in the univariable analysis, only the RI was retained in the final multivariable model. This implies that the other assessed factors could have been confounding factors and highlights the possible role of the RI as an independent predictor of cognitive group.

In addition to the memory and recognition results, some relationships were observed between the CADES scores and performance on the detour and problem box tasks. The CADES scores were positively correlated with increased DT+ and altered touching behavior. Unlike the negative association reported in a cylinder task [13], the elevated DT+ together with the increase in the CADES score may reflect intra-individual variability and perseveration rather than improved executive function. Age-related cognitive decline often manifests as episodic dysfunction [33] and behavioral inflexibility rather than isolated inhibitory failure [34]. Thus, an increased DT+ may indicate random path selection or cognitive perseveration reflecting compromised executive flexibility rather than a strategic detour approach. Altered touching behavior in the problem box task may similarly reflect changes in problem-solving strategy with age, though this association has not been consistently reported in previous studies [12]. These assessments are not precisely domain-specific but may reflect broader cognitive abnormalities in social attention, executive function, and problem-solving reported in early-stage canine cognitive dysfunction [35,36,37]. However, none of these measures were sufficient as independent diagnostic tools, and larger sample sizes are needed to corroborate these findings.

Consistent with the hypothesized relationship between neurotrophic support and short-term memory [19,38], serum BDNF concentrations were inversely associated with performance on DMTS tasks with shorter delays (3 s and 6 s). However, binary logistic regression of serum BDNF did not distinguish mildly cognitively impaired dogs from normal controls. The lack of predictive significance may be attributable to our sample having mild illness, and localized neuropathological changes may not have been sufficiently severe to elicit detectable systemic changes in circulating BDNF. Second, the small sample size may have reduced the statistical power to detect modest differences in biomarkers between groups. This is in line with Isaka et al. (2022), who reported that circulating BDNF levels often fail to consistently discriminate cognitive status with aging [22]. Thus, although BDNF may be involved in certain memory processes, its usefulness as a single biomarker to identify early-stage impairment is restricted in this sample. However, beyond these biomarker limitations, various aspects of this study need to be considered.

A major aspect of our work is the multidomain strategy, which enabled us to identify specific behavioral paradigms with discriminative relevance for mild cognitive impairment, rather than merely applying a broad cognitive test battery. Importantly, the inclusion of untrained dogs increases the clinical relevance of our results, as it more closely resembles the real-world setting of veterinary practice. The 20 s DMTS delay and neophilia-based recognition task constitute a realistic, low-intensity training measure that may be used for early cognitive screening in aging dogs. These features underscore the translational potential of targeted behavioral evaluation as an easily accessible tool for the early diagnosis of canine cognitive impairment.

The present study offers useful information for early cognitive assessment in aged dogs; however, some methodological limitations should be considered. First, only aged dogs with normal cognition or mild cognitive impairment were assessed for cognitive function. Incorporating dogs across a broader spectrum of cognitive impairment may provide a more comprehensive understanding of how cognitive test performance relates to disease severity. Second, cognitive classification depended on the CADES questionnaire, an owner-reported tool that may be subject to reporting bias. Future studies incorporating objective neuroimaging or biomarker-based confirmation would further strengthen the validity of group classification. Third, the cross-sectional design of the present study does not allow for definitive conclusions to be made about the trajectory of cognitive decline over time, and, thus, longitudinal studies are needed to fully describe the progression of early-stage impairment. Furthermore, behavioral scoring was conducted by a single examiner, which precluded assessment of inter-rater reliability; future studies should include multiple raters to improve the objectivity of behavioral evaluation. Fourth, our sample was small and homogenous, which may limit the generalizability of our findings to the larger aging dog population, and larger, more diverse cohorts are needed to validate these results. Additionally, the increased risk of false-positive results (Type I error) due to multiple hypothesis testing represents an important limitation of this study. We therefore provide FDR-adjusted results in the Supplementary Materials (Supplementary Table S1). Given the exploratory and hypothesis-generating nature of this pilot study and the small sample size (*n* = 21), rigorous multiplicity correction resulted in no variables reaching statistical significance after FDR adjustment (q < 0.05). This should be viewed in light of the lack of statistical power to detect effects given the large number of comparisons performed and not as definitive evidence of an absence of association. Nonetheless, unadjusted findings for potentially informative behavioral indices such as the 20 s DMTS task and the recognition index warrant continued attention despite not surviving correction, given the limited power inherent to this pilot design. Notably, the RI remained significant in the multivariable model after adjusting for confounders (sex, age, and body weight), in addition to the unadjusted univariable result. However, both the unadjusted univariable results and the multivariable model should be interpreted with due caution, as the latter may be subject to instability and overfitting given the small sample size. Future studies with larger, sufficiently powered cohorts are needed to validate these specific cognitive assessments. Lastly, this study did not establish diagnostic cut-off values or evaluate the sensitivity and specificity of the cognitive tests; future studies with larger sample sizes should address these factors through receiver operating characteristic (ROC) curve analysis.

## 5. Conclusions

In this exploratory study, we evaluated a multidomain approach to assessment that included cognitive–behavioral testing, owner-reported questionnaires, and serum biomarker assessment. We found that specific behavioral measures were significantly associated with the cognitive group classification of aged dogs. Specifically, while both the 20 s DMTS task and the RI derived from neophilia testing exhibited notable associations with cognitive status, the RI emerged as a potential independent predictor. Additionally, the CADES was more sensitive than the CCDR in detecting subtle impairment. Serum BDNF did not differ significantly between the groups and had no predictive value for cognitive group classification, suggesting limited potential as a standalone biomarker in this clinical cohort. Our results nevertheless support the feasibility of introducing select behavioral tasks for examining untrained, client-owned dogs in clinical settings. Importantly, our results relate specifically to dogs with mild cognitive impairment (MCI) and do not represent the full spectrum of moderate-to-severe cognitive dysfunction. Additionally, these findings should be interpreted with caution. Future validation of these tools, establishment of diagnostic cut-offs, and further exploration of potential biological correlates of early canine cognitive decline require larger, more heterogeneous cohorts, ideally with longitudinal follow-up.

## Figures and Tables

**Table 1:** Overview of primary outcomes of cognitive tests assessing cognitive performance across six domains (exploratory behavior, communication, short-term memory, executive function, attention, and novel object recognition) in recruited dogs.

Cognitive Domain	Cognitive Test	Trial/Timing	Primary Outcome
**Exploratory** **behavior**	Exploration activity	1 min	Activity score
**Communication**	Greeting behavior	Single assessment (up to 3 calls)	Greeting score
	Retrieval	5 trials	Retrieval score
	Pointing cue Marker cue	10 trials each (after warm-up/criterion)	% of correct choices
**Short-term memory**	DMTS task (3, 6, 10, 20, 40, 60, and 80 s)	6 trials/delay; progressed if ≥4/6 correct	% of correct choices by delay DMTS score
	Five pots task	5 trials; 30-s delay	% of correct choices
**Executive function**	Cylinder task	Familiarization At least 4/5 attempts Inhibitory control ≥10 trials	% of correct choices % of dogs exhibiting
	Detour task	10 trials	% of correct choices % of dogs touching cover % of dogs touching cylinder % of trials entered from detour side
	Problem box task (Trials 1–3; T1, T2, T3)	T1 once; T2 two attempts; T3 1 min	Approach score, success score, looking at the owner/examiner score, orientation score, % of dogs touching box
	Bait task	3 trials (90°, 180°, switch)	Choice accuracy score
**Attention**	Sustained gazing	Max 60 s	Sustained gaze duration
**Novel object** **recognition**	Neophilia	Neophilia: 2 trials; 60 s	Recognition index Time spent with novel object Time spent with all objects Neophilic behavior
	Neophobia	Neophobia: 1 trial; 2 min	Object interest time Object interaction time Object avoidance time

DMTS, delayed match to sample.

**Table 2:** Outcome definitions and scoring criteria for cognitive behavior test (grouped by cognitive domain).

Cognitive Domain	Test(s)	Outcome Definitions/Scoring Criteria
**Exploratory** **behavior**	Exploration activity	Score 1: <10% of the 1-min observation spent moving and floor sniffing; score 2: 10–50%; score 3: 50–90%; score 4: >90%.
**Communication**	Greeting behavior	Score 1: no approach; score 2: approach after the crouched call; score 3: approach after the repeated call; score 4: immediate approach after the initial call.
	Retrieval task	Score 1: no interest; score 2: touched the ball; score 3: bit the ball; score 4: bit the ball and returned 1–2 times; score 5: bit the ball and returned >2 times. Returned = brought the ball within the examiner’s reach.
	Pointing cue Marker cue	Not scored; outcome = % of correct choices, defined as first nose touch to the baited option.
**Short-term memory**	DMTS	Not scored; outcome = % of correct choices at each delay (first nose touch to the baited option); progression criterion: ≥4/6 correct per delay.
	DMTS score	Score 1: <20 s; score 2: 20–60 s; score 3: >60 s (maximum delay passed with ≥4/6 correct).
	Five pots task	Not scored; outcome = % of correct choices (first nose touch). Reward location pseudo-randomized (no repeats within 5 trials).
**Executive** **function**	Cylinder task	Not scored; inhibitory control: % of successful food retrieval; cylinder touches (frequency); % of dogs exhibiting inhibitory control = 0 touches. Side of approaching: % of trials approached from predefined side.
	Detour task	Not scored; outcomes = % of trials approached from predefined side; % of dogs touching cover; % of dogs touching cylinder; % of trials entered from detour side.
	Problem box task	Trial 1 (motivation for food): score 1: approached; score 2: did not approach.
		Trial 2 (food obtainable): score 1: obtained; score 2: not obtained (two attempts).
		Trial 3 (looking at the owner and looking at the examiner): score 1: never; score 2: once; score 3: twice; score 4: ≥3 times. Orientation to box: score 1: <50% of 1 min; score 2: ≥50%. Not scored: % of dogs touching box.
	Bait task (90°, 180°, switch)	Score 1: incorrect choice; score 2: correct choice. Recorded per trial.
**Attention**	Sustained gazing task	Not scored; outcome = sustained gaze duration, maximum 60 s.
**Novel object** **recognition**	Neophilia task	Not scored; outcomes = recognition index (RI *), time with novel object(s), total object interaction time (s), neophilic behavior (% of dogs).
	Neophobia task	Not scored; outcomes = object interest time (gaze time, s), object interaction time (s), object avoidance time (s).

* RI (recognition index) = time exploring novel object/total exploration time; s = second.

**Table 3:** The clinical data, cognitive questionnaire scores, and serum BDNF levels of twenty-one recruited dogs categorized into normal cognition and cognitive impairment groups according to the CADES.

	Cognitively Normal Dogs According to CADES (*n* = 9)	Cognitively Impaired Dogs According to CADES (*n* = 12)	*p*-Value
Age (months)	127.33 (96–162)	127.58 (96–168)	0.985
Sex	Male (4/9) Female (5/9)	Male (3/12) Female (9/12)	0.406
Body weight (kg)	6.4 (2.8–14)	8 (4.5–23)	0.312
CADES score	0 (0–4)	12.5 (8–23)	<0.01 ***
CCDR score	18 (16–24)	20 (16–36)	0.114
Serum BDNF (*p*g/mL)	5.10 (0.37–23.20)	5.75 (0.22–43.15)	0.915

Data are presented as median (range) for continuous variables and frequency for categorical variables. *** indicates statistical significance at *p* < 0.01.

**Table 4:** Descriptive statistics of outcomes of cognitive tests assessing cognitive performance across six domains (exploratory behavior, communication, short-term memory, executive function, attention, and neophilia/neophobia) in recruited dogs.

Cognitive Domain	Cognitive Test	Unit	Normal Cognition (*n* = 9)	Cognitive Impairment (*n* = 12)	*p*-Value
**Exploratory** **behavior**	Activity score of exploration	Score	Score 1: 0/9 Score 2: 0/9 Score 3: 5/9 Score 4: 4/9	Score 1: 0/12 Score 2: 4/12 Score 3: 4/12 Score 4: 4/12	0.220
**Communication**	Greeting behavior	Score	Score 1: 0/9 Score 2: 7/9 Score 3: 2/9 Score 4: 0/9	Score 1: 2/12 Score 2: 7/12 Score 3: 1/12 Score 4: 2/12	0.766
	Retrieval task	Score	Score 1: 2/9 Score 2: 3/9 Score 3: 1/9 Score 4: 2/9 Score 5: 1/9	Score 1: 5/12 Score 2: 5/12 Score 3: 0/12 Score 4: 2/12 Score 5: 0/12	0.191
	Pointing cue ^b^	% of correct choices	76.67 ± 37.08	83.33 ± 30.25	0.808
	Marker cue ^a^	% of correct choices	50.00 (40–80)	60.00 (35–97.5)	0.642
**Short-term memory**	DMTS task: 3 s ^b^	% of correct choices	77.78 ± 37.27	86.11 ± 29.16	0.862
	DMTS task: 6 s ^a^	% of correct choices	100 (83.33–100)	66.67 (45.83–100)	0.508
	DMTS task: 10 s ^a^	% of correct choices	100 (83.33–100)	50.00 (0–83.33)	0.082
	DMTS task: 20 s ^a^	% of correct choices	100 (66.67–100)	0 (0–79.16)	0.034 **
	DMTS task: 40 s ^a^	% of correct choices	66.67 (50–91.67)	0 (0–83.33)	0.219
	DMTS task: 60 s ^a^	% of correct choices	66.67 (0–100)	0 (0–95.83)	0.395
	DMTS task: 80 s ^a^	% of correct choices	66.67 (0–100)	0 (0–74.99)	0.219
	Score on DMTS task	Score	Score 1: 2/9 Score 2: 1/9 Score 3: 6/9	Score 1: 7/12 Score 2: 1/12 Score 3: 4/12	0.255
	Five pots task	% of correct choices	60 (40–70)	30 (20–60)	0.277
**Executive** **function**	Cylinder task: side of approach ^b^	% of correct choices	75.56 ± 28.83	74.17 ± 30.29	0.917
	Cylinder task: inhibitory control trial ^a^	% of dogs exhibiting	20(0–35)	30 (0–47.5)	0.554
	Detour trial: side of approach ^a^	% of correct choices	70 (60–85)	60 (40–85)	0.136
	Detour trial: touching cover ^a^	% of dogs exhibiting	50 (30–75)	50 (17.5–60)	0.716
	Detour task: touching cylinder ^a^	% of dogs exhibiting	50 (20–75)	55 (35–87.5)	0.538
	Detour task: enter from the detour side ^a^	% of correct choices	40 (20–85)	45 (0–77.5)	0.464
	Problem box task: Food-obtainable trial	Score	Score 1: 2/9 Score 2: 7/9	Score 1: 5/12 Score 2: 7/12	0.361
	Problem box task: block trial (looking at the owner)	Score	Score 1: 5/9 Score 2: 3/9 Score 3: 0/9 Score 4: 1/9	Score 1: 11/12 Score 2: 1/12 Score 3: 0/12 Score 4: 0/12	0.056
	Problem box task: block trial (looking at experimenter)	Score	Score 1: 6/9 Score 2: 1/9 Score 3: 2/9 Score 4: 0/9	Score 1: 8/12 Score 2: 3/12 Score 3: 1/12 Score 4: 0/12	0.831
	Problem box task: block trial (orienting toward the box)	Score	Score 1: 2/9 Score 2: 7/9 Score 3: 0/9 Score 4: 0/9	Score 1: 6/12 Score 2: 6/12 Score 3: 0/12 Score 4: 0/12	0.206
	Problem box task: touching the box	% of dogs exhibiting	4/9 (44.44%)	4/12 (33.33%)	0.673
	Bait task: changing 90°	Score	Score 1: 7/9 Score 2: 2/9	Score 1: 9/12 Score 2: 3/12	0.885
	Bait task: changing 180°	Score	Score 1: 5/9 Score 2: 4/9	Score 1: 12/12 Score 2: 0/12	0.174
	Bait task: switching position	Score	Score 1: 9/9 Score 2: 0/9	Score 1: 12/12 Score 2: 0/12	1.000
**Attention**	Sustained gazing task	Second	17.41 ± 14.67	14.62 ± 8.29	0.590
**Neophilia**	Recognition index ^a^	Proportion of dogs spending time with a novel object	0.76 (0.56–1)	0.26 (0–0.67)	0.031 **
	Time spent with novel object ^a^	Second	2.90 (1.71–5.4)	1.26 (0–2.68)	0.053
	Time spent with all objects ^a^	Second	4.23 (2.95–4.91)	3.90 (2.26–6.61)	0.696
	Neophilic behavior	% of dogs exhibiting	7/9 (77.78%)	4/12 (33.33%)	0.080
**Neophobia**	Object interest time ^b^	The total time the dog spent gazing at the toy	17.33 ± 7.42	18.83 ± 7.43	0.670
	Object interaction time ^a^	The time the dog spent interacting with the toy	23.1 (19.87–37.17)	21.95 (14.76–63.5)	0.808
	Object avoidance time ^a^	The time the dog spent moving away from the toy	76.9 (69.23–85.27)	78.05 (45.5–85.53)	0.808

Data are presented as ^a^ median (IQR) and ^b^ mean ± SD; ** indicates statistical significance at *p* < 0.05.

**Table 5:** Results of univariable binary logistic regression analysis of cognitive group classification (normal cognition or cognitive impairment group) according to serum BDNF levels and cognitive test outcomes across six cognitive domains in recruited dogs.

Cognitive Performance Variable	OR	95% CI	*p*-Value
*log*BDNF serum level	0.726	0.407–1.295	0.278
Activity level of exploration	0.407	0.109–1.524	0.182
Greeting behavior	1.051	0.331–3.339	0.933
Retrieval task	0.597	0.279–1.276	0.183
Pointing cue	1.007	0.980–1.034	0.638
Marker cue	1.007	0.981–1.033	0.623
DMTS task: 3 s	1.008	0.981–1.037	0.554
DMTS task: 6 s	0.994	0.971–1.017	0.604
DMTS task: 10 s	0.984	0.961–1.007	0.175
DMTS task: 20 s	0.976	0.953–1.000	0.048 *
DMTS task: 40 s	0.983	0.962–1.004	0.119
DMTS task: 60 s	0.988	0.969–1.007	0.215
DMTS task: 80 s	0.985	0.965–1.006	0.160
Score in DMTS task	0.441	0.162–1.196	0.108
Five pots task	0.982	0.952–1.013	0.249
Cylinder task: enter from the right side	0.998	0.968–1.030	0.911
Inhibitory control: touching the cylinder	1.014	0.979–1.049	0.438
Detour task: approaching the cover	0.967	0.923–1.012	0.148
Detour task: touching the cover	0.994	0.967–1.023	0.700
Detour task: touching a cylinder	1.009	0.982–1.037	0.517
Detour task: enter from the detour side	0.995	0.972–1.018	0.652
Problem box: obtained food	0.400	0.057–2.800	0.356
Problem box: looking at the owner	0.147	0.013–1.629	0.118
Problem box: looking at the experimenter	0.773	0.238–2.509	0.668
Problem box: orient toward the problem box	0.286	0.041–1.981	0.205
Problem box: touching the box	0.625	0.105–3.707	0.605
Bait task: 90°	1.167	0.151–9.006	0.882
Bait task: 180°	0.250	0.034–1.863	0.176
Sustained gaze	0.970	0.913–1.031	0.327
Neophilic behavior	0.143	0.020–1.032	0.054
Recognition index	0.038	0.002–0.761	0.032 *
Time spent with novel object	0.587	0.327–1.054	0.074
Time spent with all objects	1.070	0.760–1.505	0.699
Object interest time	1.028	0.910–1.162	0.652
Object interaction time	1.018	0.977–1.061	0.394
Object avoidance time	0.982	0.942–1.024	0.394

Asterisks indicate statistical significance: * *p* < 0.05.

## Data Availability

Data are available upon request.

## Supplementary Materials

The following supporting information can be downloaded at https://www.mdpi.com/article/10.3390/ani16172737/s1. Supplementary Material S1: Detailed of cognitive behavioral tests. Table S1 Univariable logistic regression results with FDR correction.
