# Supplementary material for: Concurrent Multidomain Cognitive Assessment and Serum BDNF Level in Aged Dogs: Comparison Between Normal Cognition and Mild Cognitive Impairment
Source: Animals (Basel). 2026 Sep 2;16(17):2737. doi: 10.3390/ani16172737 (PMC13565028; doi:10.3390/ani16172737)
Supplement: Supplementary file 1 [file animals-16-02737-s001.zip › animals-4431894-supplementary.pdf]

Supplementary Material

Supplementary material S1 (S1): Detailed of cognitive behavioral tests

S1. Exploratory behavior

This test aims to assess the exploratory behavior of dogs by observing the dog's activity while staying in the examination room without interaction. This test can be performed along with the habituation phase.

The activity of exploration in each dog was scored following the percentage of the dog's moving and floor sniffing within one minute.

These scores were defined as a dog's activity less than 10% (Score 1), between 10-50% (Score 2), between 50-90% (Score 3), and more than 90% (Score 4), respectively.

S2. Communication test

S2.1. Greeting behavior

This sub-test will be performed after finishing the habituation phase. The goal is to assess the outcome of dog-examiner interactions with unfamiliar people. Before starting the test, the dog and pet's owner stand at the waiting point, and the examiner stands at the testing point. Then, the examiner will call the dog's name and wait for 3 seconds to observe the dog's response. If the dog is immediately approached, the examiner had to walk closer and make a relationship with the dog by giving a reward. In case the dog is not immediately approached after calling, the examiner had to call the dog's name again in an encouraging manner. If dogs still did not show approaching, the examiner had to call with a crouch to the dog and wait for the dog's response. In case of dog show fearful behavior and not approaching, the examiner should not walk closer to the dog and wait for 10 seconds to finish the trial process.

Scoring of this test was defined following examiner behavior to dog's approach behavior, including no approaching (Score 1), approach after the crouched call (Score 2), approach after the repeated call (Score 3), and immediate approach after calling (Score 4), respectively.

S2.2. Retrieval task

The goal of this sub-test is to assess dog's willingness to perform activity with examiner and communicate behavior. Start with the examiner standing far from the pet's owner and dog. Then, examiner release rubber ball onto mat in the direction of the dog and observe retrieval behavior.

Five trials were performed, and the retrieval score was assigned into five scores: score 1: The dog is not interested in the rubber ball, score 2: The dog touched the rubber ball, score 3: The dog bit the rubber ball, score 4: The dog bit the rubber ball and returned it to the examiner 1-2 times. score 5: The dog bit the rubber ball and returned it back to the examiner more than 2 times. Returned is defined as the dog brought the ball within the examiner's reach.

S2.3. Pointing cue and marker cue

These subtests focus on dog communicative conditions and the examiner by finding a treat under an unknown cup by observing the non-verbal gesture of the examiner and marker at the cup. These cups were prepared for sub-test which are two identical cups with the same color. Before start subtests, Warm-up phase which are three phases was performed for familiarization the dog.

Briefly, Phase 1: When the examiner called the dog's name and raising the treat under dog's vision, pet's owner held the dog at waiting point. After placing treat on the mat center and saying "ok", pet's owner released the dog to approach the treat. The dog had to approach the treat at least 4 times before moving to the next phase.

Starting with phase 2, the dog was familiarized with the cup by the examiner, who hid the treat under one cup while the dog is seeing. After the examiner say "ok", pet's owner released the dog to approach the cup with treat. When the dog's nose tip touches the correct cup, examiner opened the cup and allowed the dog to assess the reward. The dog had to approach the correct cup at least 4 times in each side of cup locations (Left side and right side).

In the last phase, the recruited dog had to approach the correct cup, which had hidden food at least 4/5 times continuously from two identical cups. If the dog chose the correct cup, the examiner had to open the cup and allow the dog to approach the treat. In case the dog chose the wrong cup, the examiner opened the cup to allow the dog to see the empty cup and brought the dog to the waiting point. The sides of the cup with the treat were changed in every round of testing. The maximum trial of this phase is 20 times. Only the recruited dog was allowed to continue with pointing cues and marker cues.

For these sub-tests, the treat was hidden under one cup while covered with an opaque board to prevent the dog's vision. After that, the examiner performed a non-verbal gesture to lead the dog to approach the correct cup. Pointing cues were performed by pointing the finger to the correct cup, and marker cues were performed by placing the square box in front of the cup. Each subtest was performed 10 times separately. The percentage of correct choices was recorded for pointing cues and marker cues sub-tests that indicated the communication performance in each recruited dog.

S3. Short-term memory

S3.1. Delayed match to sample (DMTS) task

For the DMTS task, the method was performed similarly to phase 3 of the warm-up phase. The difference is the recruited dog was delayed in the time to approach the correct cup to evaluate the short-term memory. The pet's owner held the dog until the examiner said "ok". After that, the dog was allowed to approach the correct cup.

The delayed times in each trial are 3, 6, 10, 20, 40, 60, and 80 seconds. The number of trials in each delayed time is 6 trials. The recruited dog that chose the correct cup at least 4/6 times could pass to the next delayed phase. In contrast, the dog that failed the requirement of this task was not allowed to continue the task.

The average percentage of correct choice in delayed time was calculated. In addition, the score of the DMTS task was determined following the maximum delayed second that the dog reached, including score 1 (< 20 seconds), score 2 (20-60 seconds), and score 3 (> 60 seconds).

S3.2. Five pots task

This sub-test was performed by hiding a treat in one of five identical pots and delaying the time of the dog's approaching. The number of trials is five trials following the number of pots. The location of the hidden treat in each pot was randomly changed every trial without repeating pots. However, the locations of the pot were not changed throughout testing. All pots were placed on the center of the mat with a semicircular arrangement in front of the dog.

The delayed time of this sub-test is 30 seconds. The average percentage of correct choice was calculated following the correct pot that the dog correctly approached.

S4. Executive function

S4.1. Cylinder task

This sub-test is one of the executive function assessments that focus on inhibitory control and cognitive flexibility. The recruited dog had to approach food at the center of a horizontal cylindrical object via an opening. There are three trials for this task: a familiarization trial, an inhibitory control trial, and a detour trial.

For the familiarization trial, the examiner placed an opaque cylinder in the center of the mat while the pet's owner held the dog at the waiting point. Then, the examiner raised the food to the dog's vision and called the dog's name. After that, the examiner placed the food within the cylinder and said "OK" to allow the owner to release from waiting point. The dog had to investigate and assess the food within the cylindrical object at least 4/5 times continuously before moving to the next trials. The dog will be familiarized with a cylindrical object by this trial.

Next is the inhibitory control trial. The opaque cylinder was changed into a transparent cylinder to allow the dog's vision. The examiner performed the same procedure with a familiarization trial together with observing the dog's inhibitory control behavior, including the correct side of approaching (the food's placing side) and touching cylinder behavior. This trial was performed at least ten times. The recruited dog who showed cylinder touching indicated that there is no inhibitory control behavior. These records were calculated into the average percentage of correct choice and percentage of dogs exhibiting inhibitory control (defined as 0 touches).

For detour trials, the dog also approached the food within a transparent cylinder as inhibitory control. However, the cylinder opening side, which the examiner placed the food under the dog's vision, was hindered by an opaque cylinder cover for observing the inhibitory control and cognitive flexibility of the dog. this trial was performed 10 times for calculation later. In this trial, approaching the cover (the food's

placing side), touching at the cover, touching at the cylinder, and entering the correct sides (detour side) were recorded and were calculated into the average percentage of correct choice and percentage of dog exhibiting. After the cylinder task was finished, the dog was allowed to rest for at least 15 minutes and continue to the next task.

S4.2. Problem box task

There are three trials of this sub-test, which include motivation for food (Trial 1), food-obtainable trials (Trial 2), and block trials (Trial 3). A transparent box with a lid was used for the problem box task. Briefly, a transparent box without a lid was placed at the center of a mat while the owner held the dog at the waiting point. Then, the examiner showed the food to the dog's vision, called the dog's name, and placed the food into the box. After that, the examiner said "OK," and the owner released the dog to assess the food. This trial was performed in one trial, and the exhibiting behavior was scored following the dog's approach: score 1 (approach) and score 2 (not approach).

For trial 2 and trial 3, all procedures were performed similarly to trial 1, except the box was covered with a lid without locking in trial 2 and with locking in trial 3. the food-obtainable trial was evaluated from two attempts and was scored as: score 1 (obtainable) and score 2 (unobtainable). Whereas the block trial evaluated the score following the dog's behavior, including the dog's looking at the owner, looking at the experimenter, and orienting toward the problem box. For the dog's looking behavior, the score was defined as score 1 (never look), score 2 (once), score 3 (twice), and score 4 (3 times or more), while orienting toward the problem box was defined as score 1 (less than 50%) and score 2 (50% or more) and this trial was performed for 1 minute. In addition, the dog's number that touched the box was counted to calculate the percentage of dog's exhibiting with touching the box later.

S4.3. Bait task

This sub-test evaluated the dog's performance in searching for food under the cup when changing from the same position. Before starting a task, a single training session was performed to familiarize the dog with the cup by hiding the food under one cup. All dogs had to pass this training before moving to the next task. For the bait task, the examiner hid the food under one of two cups. Then, the pet's owner released the dog to select the correct cup after the examiner changed the cup position. There are three trials following the level of cup position alteration: changing 90°, changing 180°, and switching the position. The scores of these trials were score 1 (incorrect choice) and score 2 (correct choice).

S5. Attention

S5.1. Sustained gazing task

The goal of this sub-test is to assess the dog's inattentiveness by using the video camera to record the dog's gazing, which is baited with the food. Before starting the sub-test, the pet's owner held the dog at the waiting point, and the examiner prepared the video camera and carried the food. The procedures of this task are the examiner calls the pet's name, shows the food to the dog's vision, and starts to record the video. When the pet's owner heard the dog's name from the examiner, the owner released the dog to show attentiveness to the food. While the video was recorded, the dog had to continuously gaze at the baited food. The dog was not allowed to receive this food until finishing the task. The video was finished recording when the dog did not gaze at the food anymore or complete the sustained gazing until it reached 60 seconds. The average duration of sustained gazing (seconds) was noted for statistical analysis.

S6. Novel object recognition

S6.1. Neophilia task

This sub-test is one of the novel object recognition sub-tests that assessed the neophilia behavior of the dog when encountering a novel object. In this task, the examiner prepared the three novel objects, which are different in shape and color. Before starting the task, the pet's owner and dog stayed at the waiting point and turned around to prepare the novel object.

In the first round, the examiner placed the two novel objects at the mat's center. After placing the objects, the examiner sends the signal to the owner by saying "ok.". Then, the owner released the dog to explore the novel object for 1 minute. After one minute, the owner brought the dog back to the same position and turned around again.

In second round, the examiner had to change one of the novel objects to create the novel object recognition event. After that, the owner released the dog to explore all objects (the same object and the new object) on the mat after receiving the signal from the examiner. While the dog explored the object in the second round, the video was recorded to assess the time duration of the dog to explore this novel object and the time duration to explore all total objects.

Lastly, the recognition index was calculated from the proportion of the time duration of novel object exploration by the time duration of total object exploration. In addition, neophilic behavior was evaluated by the presence or absence of exploring novel objects before or after familiar objects.

S6.2. Neophobia task

The neophobia task focused on novel object recognition, similar to the neophilia task. On the other hand, this subtest was performed using a toy dog to assess the fear and avoidance of a dog's behavior of the novel object. This phase evaluated the dog's interaction with the toy dog while staying in the examination room alone. Before starting, the pet's owner waited outside the examination room and left the dog with the examiner. Then, the examiner started the video camera to record all the dog's behavior between tests and activated the toy dog on the mat. After that, the examiner had to leave the dog with the novel object for two minutes.

The total time of dog gazing, the duration of dog spending time for interaction (touching, biting, and smelling), and moving away (walking away and running away) were evaluated from the video record to calculate object interest duration, object interaction, and object avoidance for statistical analysis.

Supplementary table S1: Unadjusted and False Discovery Rate (FDR)-adjusted p-values for univariable logistic regression analyses

Table S1 Univariable logistic regression results with FDR correction

Variable	Raw p-value	FDR q-value (BH)	Significant (q < 0.05)
Recognition Index	0.032	0.507	N
DMTS task: 20 seconds	0.048	0.507	N
Neophilic behavior	0.054	0.507	N
Time of spending novel object	0.074	0.507	N
Score of DMTS task	0.108	0.507	N
Problem box: looking at the owner	0.118	0.507	N
DMTS task: 40 seconds	0.119	0.507	N
Detour task: approaching the cover	0.148	0.507	N
DMTS task: 80 seconds	0.160	0.507	N
DMTS task: 10 seconds	0.175	0.507	N
Bait task: 180°	0.176	0.507	N
Activity level of exploration	0.182	0.507	N
Retrieval task	0.183	0.507	N
Problem box: orient toward the problem box	0.205	0.516	N
DMTS task: 60 seconds	0.215	0.516	N
Five pots task	0.249	0.560	N
logBDNF serum level	0.278	0.589	N
Sustained gaze	0.327	0.654	N
Problem box: obtained food	0.356	0.675	N
Object interaction	0.394	0.675	N
Object avoidance	0.394	0.675	N
Inhibitory control: touching the cylinder	0.438	0.717	N
Detour task: touching a cylinder	0.517	0.764	N
DMTS task: 3 seconds	0.554	0.764	N
DMTS task: 6 seconds	0.604	0.764	N
Problem box: Touching the box	0.605	0.764	N
Marker cue	0.623	0.764	N
Pointing cue	0.638	0.764	N
Detour task: enter at the detour side	0.652	0.764	N
Object interested duration	0.652	0.764	N
Problem box: looking at the experimenter	0.668	0.764	N
Time of spending total objects	0.699	0.764	N
Detour task: touching the cover	0.700	0.764	N
Bait task: 90°	0.882	0.933	N
Cylinder task: enter the right side	0.911	0.933	N
Greeting behavior	0.933	0.933	N

Note: Following Benjamini-Hochberg FDR correction ($q<0.05$), no variables remained statistically significant, including the two variables with raw $p<0.05$ (Recognition Index, $p=0.032$; DMTS task: 20 seconds, $P=0.048$). As this is an exploratory pilot study with a limited sample size ($n=21$), these adjusted values are presented alongside unadjusted results to provide a comprehensive overview while avoiding the premature dismissal of potential behavioral markers. Abbreviations: Y, FDR-adjusted q-value <0.05 (survived correction); N, FDR-adjusted q-value ≥0.05 (did not survive correction).
